# Supplementary material for: Robust Cre-Mediated Recombination in Small Intestinal Stem Cells Utilizing the Olfm4 Locus
Source: Stem Cell Reports. 2014 Jun 26;3(2):234–41. doi: 10.1016/j.stemcr.2014.05.018 (PMC4175542; doi:10.1016/j.stemcr.2014.05.018)
Supplement: Document S1. Figures S1–S4 [file mmc1.pdf]

Stem Cell Reports, Volume *3*

Supplemental Information

Robust Cre-Mediated Recombination in Small Intestinal Stem Cells Utilizing the *Olfm4* Locus

Jurian Schuijers, Laurens G. van der Flier, Johan van Es, and Hans Clevers

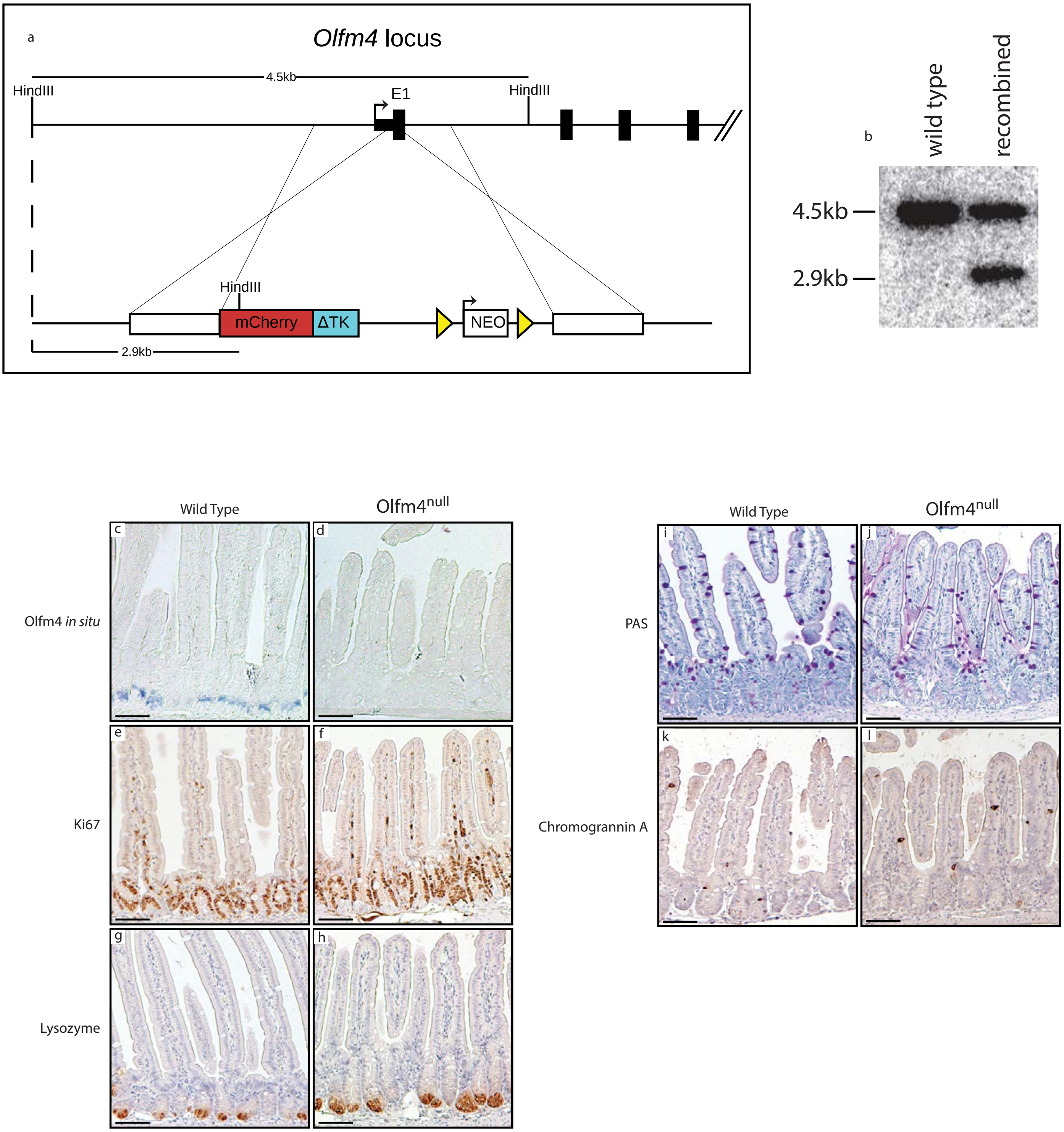
a
Olfm4 locus
4.5kb
HindIII
E1
HindIII
HindIII
mCherry
ΔTK
NEO
2.9kb
b
wild type
recombined
4.5kb
2.9kb
Wild Type
Olfm4null
Olfm4 in situ
Ki67
Lysozyme
c
d
e
f
g
h
Wild Type
Olfm4null
PAS
Chromogrannin A
i
j
k
l

Supplementary Figure 1. Olfm4^{null} animals show no intestinal phenotype. Supplementary to Figure 1.

A) Shows the targeting strategy for the generation of Olfm4-CherryΔtk knock-in allele. The CherryΔtk fusion is introduced in frame of the ATG in the first exon of the *Olfm4* locus. Neo, neomycin resistance cassette.

B) Southern blot of targeted mouse ES cells shows a homozygous wild-type allele in control lane 1 and a heterozygous-targeted allele in lane 2.

C,D) *In situ hybridization* probing for Olfm4 mRNA showing the complete loss of Olfm4 messenger in the Olfm4^{null} intestines.

E,F) Immunohistology staining for Ki67 normal proliferation in wild type and Olfm4^{null} intestines.

G,H) Immunohistology staining for lysozyme showing normal numbers and localization of Paneth cells in wild type and Olfm4^{null} intestines.

I,J) PAS staining showing normal numbers of goblet cells in wild type and Olfm4^{null} intestines.

K,L) Immunohistology staining for Chromogrannin A showing normal numbers of enteroendocrine cells in wild type and Olfm4^{null} intestines. Scale bars 100μM.

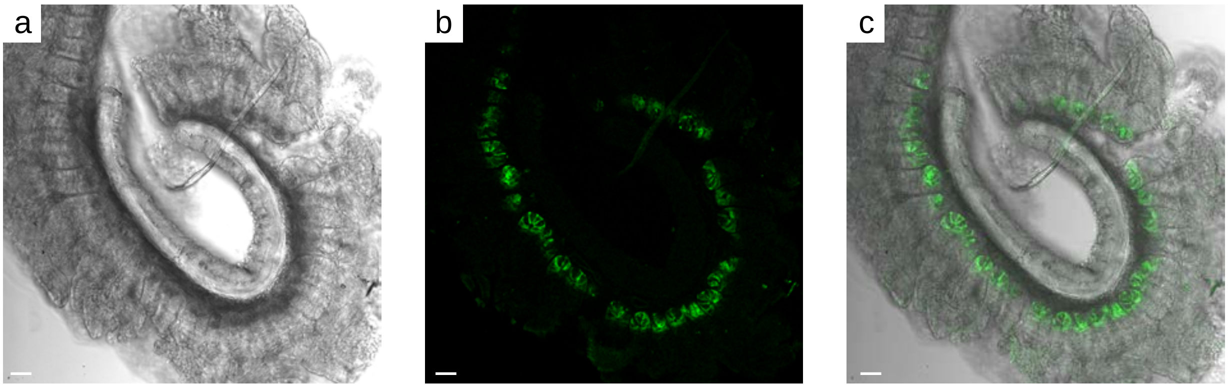
a
b
c

Supplementary Figure 2. *Olfm4-IRES-eGFPCreERT2* marks all the crypts of the small intestine. Supplementary to Figure 2.

A-C) Low magnification fluorescence microscopy showing complete penetrance of the *Olfm4-IRES-eGFPCreERT2* alleles in heterozygous animals. Scale bars 50µM.

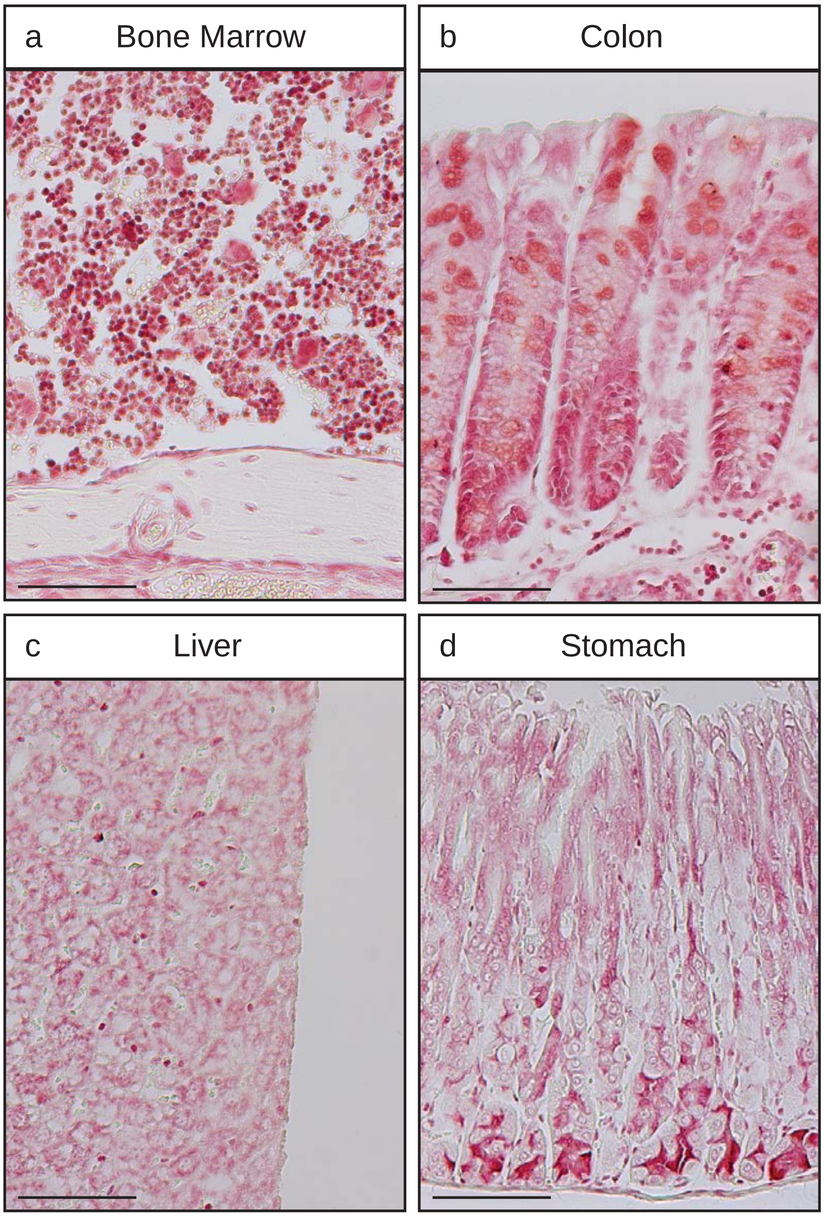
a
Bone Marrow
b
Colon
c
Liver
d
Stomach

Supplementary Figure 3. *Olfm4-IRES-eGFPCreERT2* is exclusively expressed in the small intestine. Supplementary to figure 3.

A-D) Immunohistochemistry showing representative pictures of the a) Bone Marrow, b) Colon, c) Liver and d) Stomach of tamoxifen induced heterozygous *Olfm4-IRES-eGFPCreERT2* 7 days after induction. Scale bars 50µm.

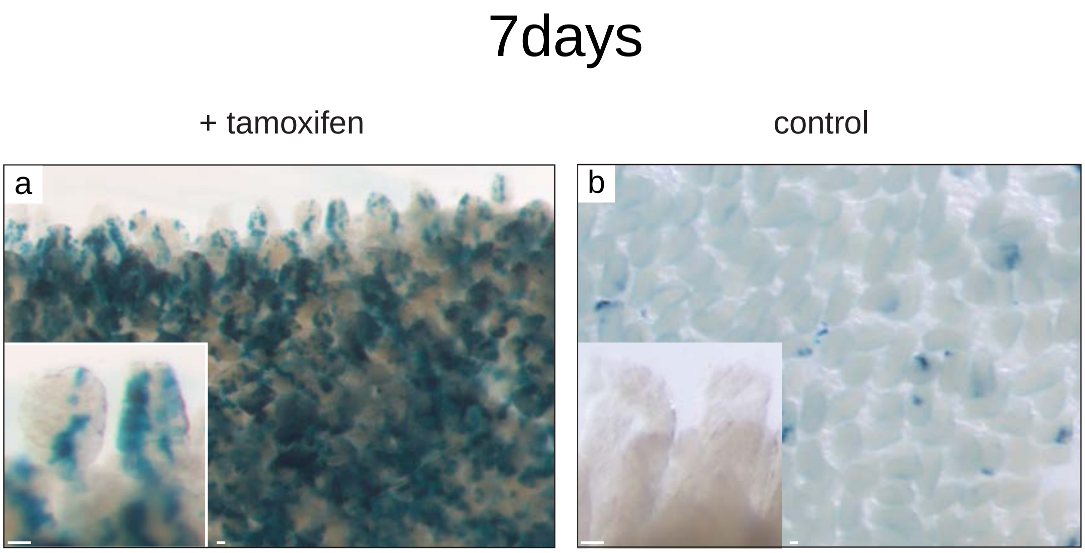
7days
+ tamoxifen
control
a
b

Supplementary Figure 4. Olfm4-IRES-eGFP-CreERT2 background induction. Supplemenary to figure 4.

A) Whole mount image showing LacZ staining of an *Olfm4-IRES-eGFPCreERT2* heterozygous animal 7 days after induction with tamoxifen.

B) Whole mount image showing LacZ staining of an *Olfm4-IRES-eGFPCreERT2* heterozygous animal without tamoxifen induction. Scale bars 50µM.
