# Supplementary material for: Robust Cre-Mediated Recombination in Small Intestinal Stem Cells Utilizing the Olfm4 Locus
Source: Stem Cell Reports. 2014 Jun 26;3(2):234–41. doi: 10.1016/j.stemcr.2014.05.018 (PMC4175542; doi:10.1016/j.stemcr.2014.05.018)
Supplement: Document S2. Article plus Supplemental Information [file mmc2.pdf]

Stem Cell Reports
Report

ISSCR
OPEN ACCESS

Robust Cre-Mediated Recombination in Small Intestinal Stem Cells Utilizing the *Olfm4* Locus

Jurian Schuijers,[1] Laurens G. van der Flier,[1,2] Johan van Es,[1] and Hans Clevers[1,*]
[1]Hubrecht Institute for Developmental Biology and Stem Cells, University Medical Centre Utrecht, University of Utrecht, Utrecht, Uppsalalaan 8, 3584CT Utrecht, the Netherlands
[2]Present address: SomantiX B.V., Utrecht, Padualaan 8, 3584 CH Utrecht, the Netherlands
*Correspondence: h.clevers@hubrecht.eu
http://dx.doi.org/10.1016/j.stemcr.2014.05.018
This is an open access article under the CC BY license (http://creativecommons.org/licenses/by/3.0/).

SUMMARY

The epithelium of the small intestine is the most rapidly self-renewing tissue in mammals. We previously demonstrated the existence of a long-lived pool of cycling stem cells defined by *Lgr5* expression at the bottom of intestinal crypts. An *Lgr5-eGFP-IRES-CreERT2* knockin allele has been instrumental in characterizing and profiling these cells, yet its low level expression and its silencing in patches of adjacent crypts have not allowed quantitative gene deletion. *Olfactomedin-4* (*Olfm4*) has emerged from a gene signature of *Lgr5* stem cells as a robust marker for murine small intestinal stem cells. We observe that *Olfm4^{null}* animals show no phenotype and report the generation of an *Olfm4-IRES-eGFPCreERT2* knockin mouse model that allows visualization and genetic manipulation of *Lgr5*+ stem cells in the epithelium of the small intestine. The eGFPCreERT2 fusion protein faithfully marks all stem cells in the small intestine and induces the activation of a conditional LacZ reporter with robust efficiency.

INTRODUCTION

Intestinal crypts contain stem cells and their transit-amplifying (TA) daughter cells. Cells exiting the proliferative crypts onto the villi terminally differentiate into enterocytes, goblet cells, and enteroendocrine cells. Paneth cells escape the crypt-villus flow by migrating to crypt bottoms, where they live for several weeks (Bjerknes and Cheng, 2006). With the exception of stem cells and Paneth cells, the murine small intestinal epithelium is renewed approximately every 5 days (van der Flier and Clevers, 2009).

About 14 *Lgr5*+ stem cells reside intermingled with the Paneth cells at the very bottom of the crypts, where they divide and give rise to all the cell types mentioned above (Barker et al., 2007; Snippert et al., 2010). A second pool of long-lived, label-retaining cells has been postulated to exist at a position directly above the Paneth cells (Potten, 1977; Potten et al., 1974). These so-called +4 cells express markers such as *Bmi1* (Tian et al., 2011), *Lrig1* (Powell et al., 2012), and *HopX* (Takeda et al., 2011). Paradoxically, *Lgr5*+ stem cells also express these markers (Muñoz et al., 2012). A recent study reconciled these findings by showing that noncycling Paneth/enteroendocrine cell precursors coexpress *Lgr5* and the +4 markers, and can revert to an *Lgr5*-stem cell phenotype upon damage (Buczacki et al., 2013; Muñoz et al., 2012; reviewed in Clevers, 2013).

Intestinal stem cells were identified and initially characterized with the use of an *Lgr5-eGFP-IRES-CreERT2* allele (Barker et al., 2007). This model has proved to be very useful for such studies, but selective silencing of the mutant allele consistently leads to a mosaic expression of the GFP and CreERT2 proteins in patches of crypts. Silencing is limited in the duodenum but is rather extensive in the distal small intestine. Homozygotes of this model cannot be used because of the perinatal mortality of *Lgr5^{null}* pups (Morita et al., 2004). Additionally, studies have described *Lgr5-DSRED-IRES-CreERT2* and *Lgr5-DTReGFP* alleles (Tian et al., 2011) that make use of the specific expression pattern of *Lgr5*. However, these two models also abolish *Lgr5* expression, preventing the generation of high-marker-expressing homozygous animals. Furthermore, the expression levels of *Lgr5* are very low, which makes it challenging to use alternative techniques, such as in situ hybridization and immunohistochemistry, to visualize the stem cells (Kemper et al., 2012; Tian et al., 2011).

We previously generated a differential gene-expression profile for *Lgr5* stem cells and their immediate daughters by GFP-based sorting of epithelial cells from isolated crypts of *Lgr5-EGFP-ires-CreERT2* mice. When expression of individual genes was tested by in situ hybridization analysis, *Olfm4* emerged as a highly specific and robust marker for *Lgr5* stem cells. The highly stem cell-specific expression pattern of *Olfm4* was also confirmed by single-molecule fluorescent in situ hybridization (Itzkovitz et al., 2012) and mass spectrometry (Muñoz et al., 2012). Although *Olfm4* was not expressed in murine colon, human *OLFM4* has been found to be enriched in both small intestinal and colonic crypts, as well as in subsets of colorectal carcinomas (van der Flier et al., 2009a).

The *OLFM4* gene was originally cloned from human myeloblasts. It encodes for a 54 kDa protein of unknown function, which was predicted to be secreted (Zhang et al., 2002). Subsequently, it was shown that *Xenopus ONT1*, an Olfactomedin family member, acts as a BMP

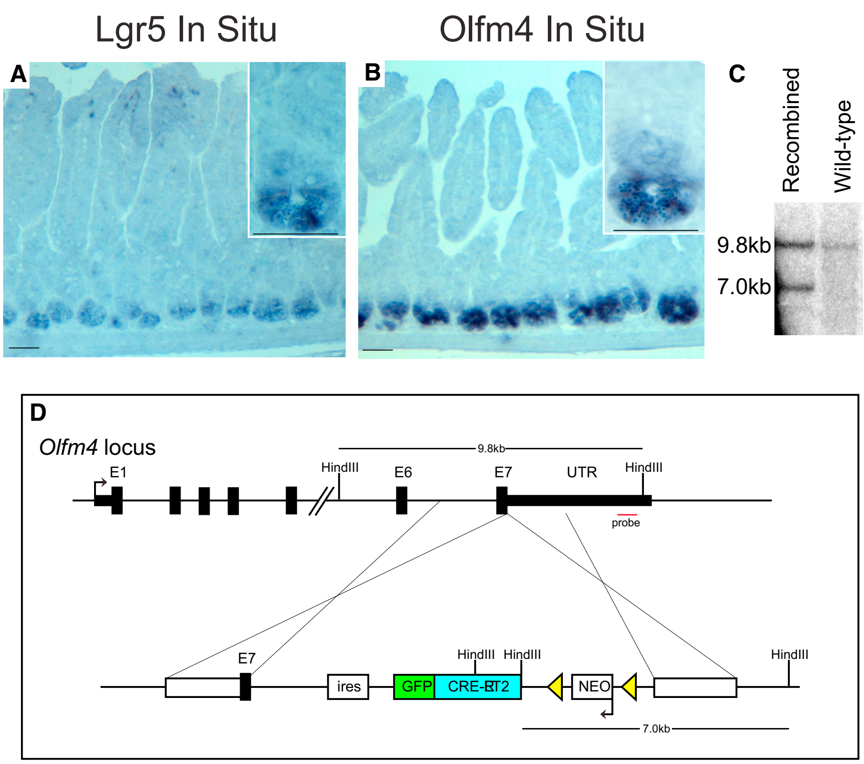

Figure 1. *Olfm4* Expression Is Restricted to the Stem Cells in the Small Intestine

(A) In situ hybridization with a probe for *Lgr5*. *Lgr5* mRNA is restricted to the stem cells between the Paneth cells at the bottom of the crypt. Scale bars, 50 μm.

(B) In situ hybridization with a probe specific for *Olfm4* mRNA. *Olfm4* expression is restricted to the same cells that also express *Lgr5*. *Olfm4* mRNA is observed in stem cells between differentiated Paneth cells at the bottom of the crypt. Scale bars, 50 μm.

(C) Southern blot of targeted mouse ESCs shows a heterozygous-targeted allele in lane 1 and a homozygous wild-type allele in control lane 2.

(D) The IRES-eGFPCreERT2 construct was cloned just after the stop codon of the last exon of *Olfm4*, making use of the endogenous poly A signal. This strategy retains the endogenous expression pattern and levels because regulatory sequences in the promoter and UTRs are maintained.

See also Figure S1.

antagonist (Inomata et al., 2008). Additionally, an *Olfm4* knockout mouse model was generated, which showed a function for *Olfm4* in repressing the immune system to facilitate sustained *Helicobacter pylori* infection (Liu et al., 2010). In this context, *Olfm4* was identified as an NFkB target. Loss of *Olfm4* has been associated with progression of prostate cancer (Chen et al., 2011; Li et al., 2013) and *Olfm4* was reported to be a Notch target in intestinal progenitor cells (VanDussen et al., 2012). Although the function and regulation of *Olfm4* within the intestinal epithelium remain to be fully elucidated, the highly specific expression pattern of this gene in intestinal crypt stem cells prompted us to generate a knockin (KI) mouse line with the aim to generate a robust tool for visualization and gene modification in small intestinal stem cells.

RESULTS

Olfm4^{null} Animals Do Not Display a Phenotype

Olfm4 was previously identified as a gene enriched in intestinal stem cells by microarray analysis after fluorescence-activated cell sorting isolation of *Lgr5*+ stem cells. Additionally, the high levels of *Olfm4* mRNA in intestinal stem cells have made it a standard marker for visualization of stem cells by in situ hybridization, as shown in previous studies (Potten, 1977; van der Flier et al., 2009a). These and our analyses showed that the expression pattern of *Olfm4* in the small intestine is remarkably similar to that of *Lgr5* (Figures 1A and 1B). *OLFM4* was also shown to be expressed in the stem cell compartment of the human small intestine, the colon, and a subset of colorectal cancers. In the mouse, it is restricted to the small intestine. We generated an $Olfm4^{null}$ allele to study the function of *Olfm4*. Homozygous animals lacking *Olfm4* mRNA were healthy and fertile, but did not show any detectable phenotype (Figure S1 available online), confirming previous findings (Liu et al., 2010). Of note, the inserted mCherry served as a roadblock, but was not expressed.

Generation of an *Olfm4-IRES-eGFPCreERT2* Allele

To visualize live *Olfm4* cells and test whether these cells are indeed intestinal stem cells, we generated a KI mouse in which an IRES-eGFPCreERT2 cassette was inserted at the stop codon located in the last exon of the *Olfm4* gene (Figure 1D). This strategy makes use of the endogenous poly A signal and 3′ UTR of the *Olfm4* gene. The *eGFPCreERT2* construct encodes for a fusion protein of eGFP and the tamoxifen-inducible CreERT2 enzyme. Thus, this fusion protein allows the visualization of *Olfm4*-expressing cells by cytoplasmatic GFP fluorescence and offers the possibility to, upon tamoxifen administration, induce and monitor Cre activation within these cells by nuclear GFP fluorescence. Because *Olfm4* was reported to encode a

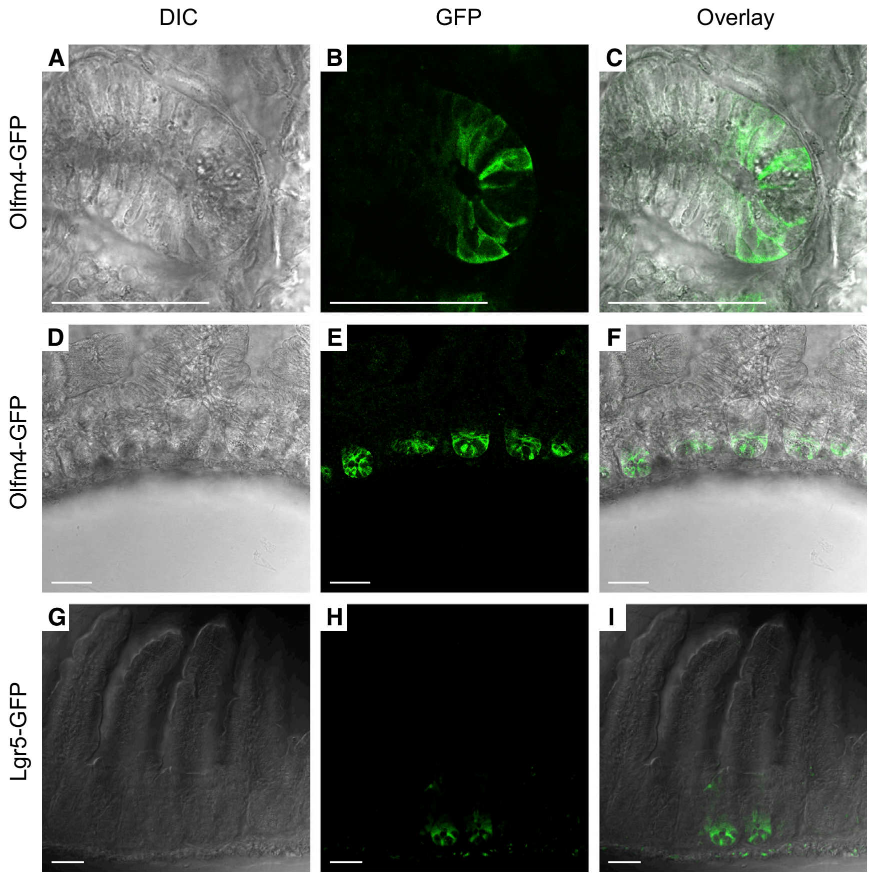

Figure 2. *Olfm4-IRES-eGFPCreERT2* Is Expressed in the Stem Cells of the Small Intestine

(A–C) High magnification showing eGFPCreERT2 fluorescence specifically in the stem cells in the bottom of the crypt, excluding the differentiated Paneth cells.

(D–F) Confocal imaging showing the eGFP fluorescence of the eGFPCreERT2 fusion protein. Fluorescence is restricted to the bottom of the intestinal crypts. Low magnification shows that eGFPCreERT2 expression is observed in every crypt of the small intestine, even in heterozygous animals. eGFP signal was magnified by anti-eGFP antibody staining.

(G–I) *Lgr5*-eGFP-IRES-CreERT2 fluorescence in the crypts of the small intestine. The expression is specific for stem cells, but in several crypts the recombined allele has been silenced. Scale bars, 50 μm.

See also Figure S2.

secreted protein product, an IRES sequence was used to prevent the fusion protein from being directed to the exocytotic pathway.

Southern blot analysis with a probe downstream of the targeted region confirmed correct homologous recombination in approximately 1 in 300 embryonic stem cell (ESC) clones (Figure 1C). Blastocyst injection yielded multiple chimeric mice. Heterozygous and homozygous mice were retrieved at the expected Mendelian ratios at birth. Adult transgenic animals showed no obvious abnormalities and displayed a lifespan and fertility comparable to those of wild-type littermates.

Confocal analysis of eGFP-stained small intestines from heterozygous *Olfm4-IRES-eGFPCreERT2* adult mice revealed fluorescence localized in small intestinal crypts (Figures 2A–2C). *Olfm4*-driven GFP is expressed only in the bottom of the crypts in the slender stem cells, and not in the granulated Paneth cells. Slender GFP+ cells were observed in the bottom of all crypts of the epithelium in the small intestine, but not the colon. We identified *Olfm4* as a stem cell marker using the *Lgr5-GFP-IRES-CreERT2* mouse model. This mouse model expresses GFP in a fraction of all crypts of the small intestine (Figures 2G–2I). In contrast, *Olfm4*-driven GFP expression was found in every crypt of the small intestine (Figures 2D–2F and S2). To test whether the *Olfm4-IRES-eGFPCreERT2* allele would mark cells in vitro, we derived organoids from homozygous *Olfm4-IRES-eGFPCreERT2* animals. Organoids were cultured under previously described conditions (Sato et al., 2009) and could be maintained for at least 12 weekly passages. GFP expression was observed at the tips of budding crypts in the absence and presence of 4OH-tamoxifen (4OHT), recapitulating the GFP and *Olfm4* pattern described previously in vivo (Sato et al., 2009). Notably, eGFP was observed in the cytoplasm in the absence of 4OHT (Figures 3A and 3B). When 4OHT was added to the culture medium, GFP fluorescence completely relocalized to the cell nucleus (Figures 3C and 3D), indicating efficient nuclear translocation of the eGFPCreERT2 fusion protein.

Olfm4-Driven eGFPCreERT2 Is Specifically Expressed in the Stem Cells of the Small Intestine

To test the potential of *Olfm4*-expressing cells to serve as a stem cell reservoir of the intestinal epithelium, we crossed the *Olfm4-IRES-eGFPCreERT2* KI mice with *Rosa26-LacZ*

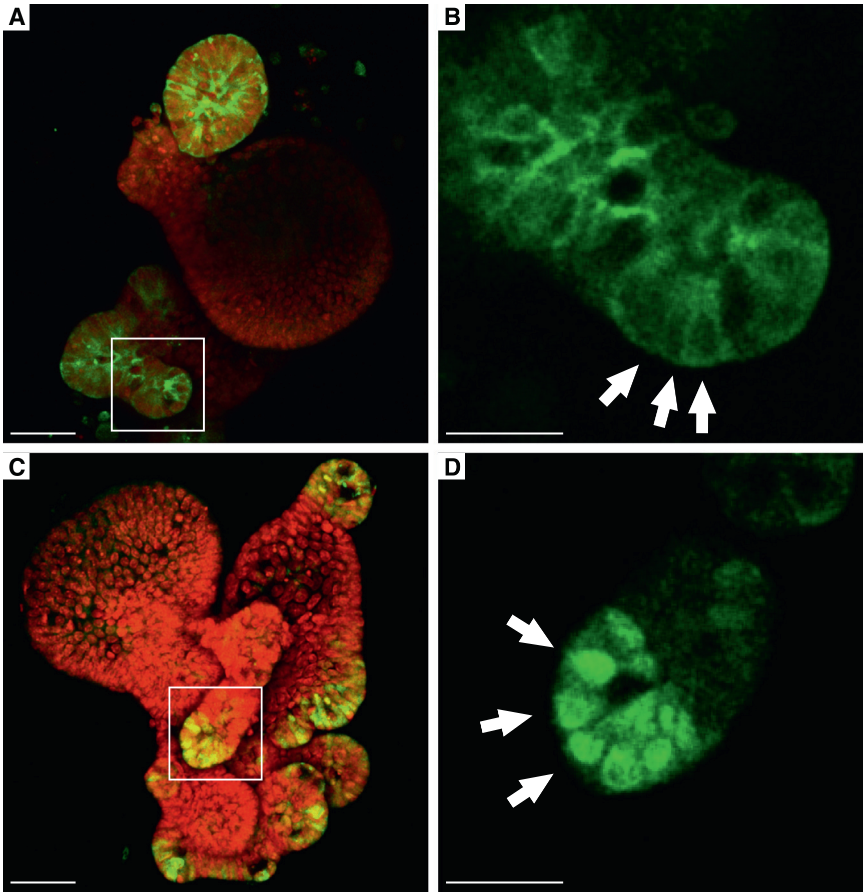

Figure 3. *Olfm4-IRES-eGFPCreERT2* Is Expressed Specifically in the Stem Cells of Intestinal Organoid Cultures

(A and B) Confocal imaging showing specific expression of the eGFPCreERT2 fusion gene in the cytoplasm of cells in the budding tips of crypt-like domains in intestinal organoid cultures. Arrows indicate cytoplasmatic eGFP fluorescence prior to 4OHT induction.

(C and D) Confocal imaging showing specific expression of the eGFPCreERT2 fusion gene in the nucleus of 4OHT-induced cells in the budding tips of crypt-like domains in intestinal organoid cultures. Nuclei were stained with TOPRO-3. The eGFP signal was magnified by anti-eGFP antibody staining. Arrows indicate nuclear eGFP fluorescence after 4OHT induction. Scale bars, 50 μm.

See also Figure S3.

reporter mice (Soriano, 1999). Upon injection of tamoxifen, the eGFPCreERT2 enzyme is activated to excise a LoxP-flanked roadblock sequence in *Rosa26-LacZ* alleles. As a result, *Olfm4*-expressing cells are genetically marked by an activated LacZ reporter. Moreover, because the process is irreversible, all progeny of *Olfm4* cells will bear the same marking, enabling tracing of the lineage over time. Adult mice were injected once with tamoxifen and LacZ staining was performed at 15 hr, 30 hr, 7 days, and 3 months after administration (Figures 4A–4E). LacZ+ cells were observed exclusively in the intestine, and not in the colon, stomach, bone marrow, or liver (Figure S3).

To visualize the exact location of *Olfm4* cells in which tracing initiated, we analyzed LacZ expression within sagittal intestinal sections. After 15 hr, LacZ+ cells appeared between Paneth cells at the crypt bottoms (Figure 4A). We quantified the positions at which LacZ+ cells appeared relative to the crypt bottoms (Figure 4H). Most LacZ+ cells were detected at positions 0, 1′, and 2′. These data were remarkably similar to the published quantifications of lineage-tracing initiation in *Lgr5-EGFP-IRES-CreERT2* KI mice (Barker et al., 2007; Figure 4H). Longer tracing experiments showed that the *Olfm4*+ cells repopulated the entire intestinal epithelium within 7 days, as has been shown for *Lgr5* stem cells (Figure 4C). These cells were able to maintain the epithelium for at least 3 months (Figure 4D). To determine whether *Olfm4-IRES-eGFPCreERT2*-expressing cells give rise to all the lineages in the intestinal epithelium, we selected crypts in which low induction efficiency caused labeling of a small number of stem cells and, as a result, individual daughter cells. We observed that *Olfm4*-expressing cells were able to generate all major intestinal cell types, namely, Paneth cells, enterocytes, enteroendocrine cells, and goblet cells (Figures 4E–4G), thus proving that *Olfm4* is a bona fide intestinal stem cell marker.

Olfm4-IRES-eGFPCreERT2-Driven DNA Recombination Is Highly Efficient

In order to maximize Cre-mediated DNA recombination, we injected mice with three daily doses of tamoxifen and compared the recombination efficiency after 7 days in mice that received a single dose or no tamoxifen. We scored approximately 1,000 crypts per condition and found that the animals that had received 1 dose of tamoxifen were expressing LacZ in 48% of the crypts (Figure 4I). In contrast, in animals that had received three tamoxifen injections,

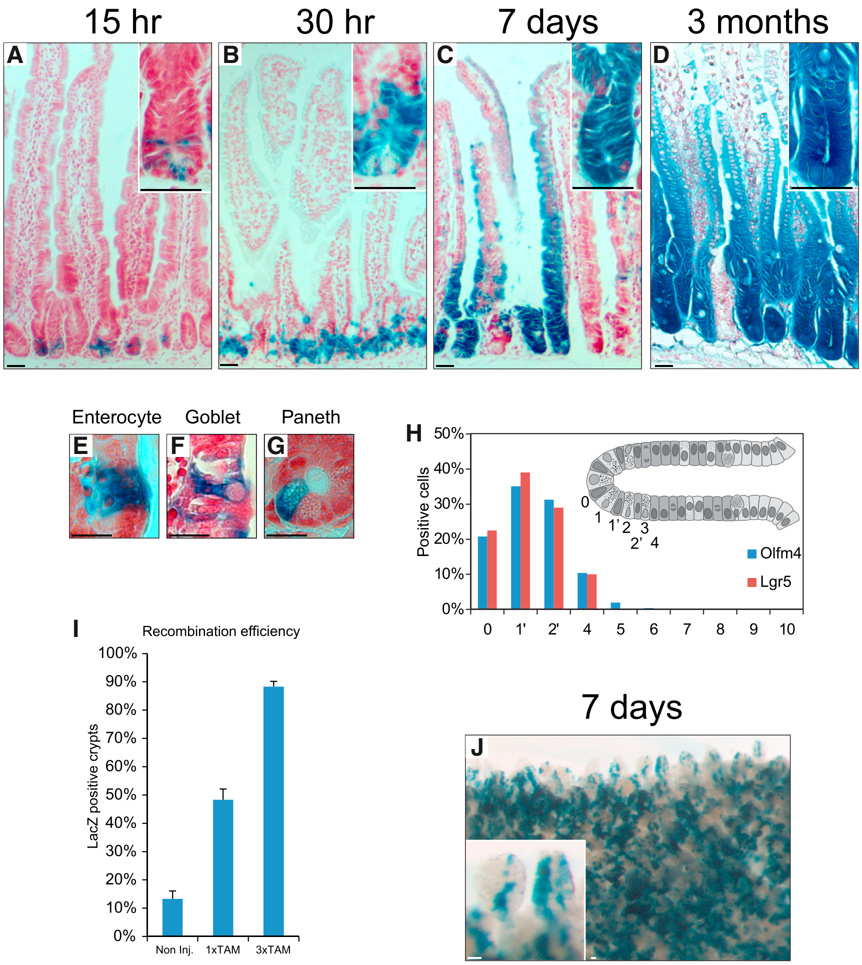

Figure 4. Lineage Tracing Using the eGFPCreERT2 Fusion Gene Shows that *Olfm4* Marks Stem Cells in the Small Intestine

(A–D) Histological analysis of LacZ activity after its induction through activation of the CreERT2 domain of the eGFPCreERT2 fusion gene. Mice were induced with a single dose of 5 mg/kg tamoxifen and then sacrificed after 15 hr, 30 hr, 7 days, or 3 months. After 15 hr, only a limited amount of cells at the bottom of the crypt are genetically labeled, whereas after 30 hr the bottom half of the crypt is completely recombined. After 7 days, the full crypt-villus axis has been renewed and consists of recombined daughter cells. After 3 months, the recombined stem cells persist and have continued to give rise to all cell types of the intestinal epithelium. Scale bars, 50 μm.

(E–G) LacZ+ cells 30 hr after tamoxifen induction show that enterocytes, goblet cells, and Paneth cells belong to the progeny of *Olfm4*+ cells. Scale bars, 25 μm.

(H) The frequency at which blue cells appeared at specific locations was counted relative to the crypt bottom 15 hr after induction of tracing. Results are depicted as the means of five independent stretches of proximal small intestine totaling >300 crypts. Most Cre LacZ-labeled cells occurred at stem cell positions 1′ and 2′, which are highly similar to the positions where *Lgr5* stem cells reside. Quantitative data on the start position of lineage tracing with the use of *Lgr5*-EGFP-IRES-CreERT2 KI mice were published previously (Barker et al., 2007). The graph shows a comparison between the initial tracing positions of both mouse models.

(I) Efficiency of Cre-mediated DNA recombination. Animals heterozygous for the *Olfm4-IRES-eGFPCreERT2* allele and carrying the R26R-LacZ reporter were induced with either three daily tamoxifen injections or one single injection, or not induced at all. Three stretches of ~330 crypts were then evaluated for LacZ reporter gene expression and the average efficiency was plotted. Error bars, SD.

(J) Whole-mount image of LacZ staining 7 days after induction of the eGFPCreERT2 fusion gene with tamoxifen. After three daily tamoxifen injections up to 88% of the crypts are recombine and LacZ expressing progeny occupies all villi of the small intestine. Scale bars, 50 μm. See also Figure S4.

88% of the crypts of the small intestine were expressing the LacZ reporter (Figures 4I and 4J). Noninjected mice showed very rare background tracing events (Figures 4I and S4).

Taken together, these results show that *Olfm4* is expressed in the stem cells of the small intestine, and the *eGFPCreERT2* KI allele allows for efficient genetic manipulation of these cells.

DISCUSSION

The availability of *Lgr5* as a specific marker for stem cells in the intestine and other tissues has allowed the unequivocal identification of intestinal stem cells, as well as molecular profiling (Muñoz et al., 2012), the establishment of culture methods (Sato et al., 2009), and the identification of signaling cascades involved in stem cell homeostasis (de Lau et al., 2011). Although the identification of *Lgr5* and the generation of the *Lgr5-GFP-IRES-CreERT2* KI mouse were pivotal for the identification of intestinal stem cells (Barker et al., 2007), the low expression levels of *Lgr5* and the mosaic expression of the first KI allele have limited *Lgr5*'s use as a marker for stem cells. For example, cell ablation studies are not possible due to the suboptimal penetrance in this particular model. *Olfm4* was reported to be a highly specific stem cell marker

(Itzkovitz et al., 2012; Muñoz et al., 2012; van der Flier et al., 2009a) and noted for the high levels of RNA in the stem cells (Muñoz et al., 2012). Recently, a pool of noncycling stem cells was identified in the pool of *Lgr5*+ crypt cells (Buczacki et al., 2013). Because of the highly similar expression profiles of *Olfm4* and *Lgr5*, it is likely that these cells are also marked by Olfm4. Here, we have described the generation of an *Olfm4-IRES-eGFPCreERT2* allele that allows the visualization of stem cells of the small intestine, as well as the genetic manipulation of these cells.

In contrast to some of the previously described mouse models (Barker et al., 2007), the *Olfm4-IRES-eGFPCreERT2* allele is expressed in all crypts of the intestine and is not silenced. In individual crypts, *Olfm4-IRES-eGFPCreERT2* is expressed in all of the stem cells that are also marked by *Lgr5*, including the label-retaining stem cells (Buczacki et al., 2013) and the so-called "border cells" (Ritsma et al., 2014). This allows for the quantitative manipulation of the entire stem cell pool. Replenishment of the intestinal epithelium occurs via a pattern of neutral drift dynamics (Lopez-Garcia et al., 2010; Ritsma et al., 2014; Snippert et al., 2010) in which "unhealthy" stem cell clones are rapidly lost. In previous studies, competition of wild-type stem cells with genetically altered stem cells made it difficult to discern phenotypes (van der Flier et al., 2009b). The complete penetrance of the *Olfm4-IRES-eGFPCreERT2* allele circumvents this problem by allowing the simultaneous alteration of a large majority of the stem cells, favoring the new genotype. In our analysis of the *Olfm4-IRES-eGFPCreERT2* allele, we found a limited activation of the Rosa-LacZ reporter in the absence of tamoxifen; however, this does not influence the usefulness of this model for cell ablation studies.

We also show the *Olfm4*-driven expression of GFP in organoid cultures derived from *Olfm4-IRES-eGPCreERT2* animals, where it is observed exclusively in the slender cells between the Paneth cells at the bottom of the crypt-like buds of the cultures. Due to the rapid expansion of the organoids in this culture model, the Paneth and stem cell domain is enlarged, and Olfm4-driven GFP expression marks the complete stem cell pool in these cultures.

In contrast to *Lgr5*, the expression of the introduced *eGFPCreERT2* fusion gene was limited to cells of the small intestine only. This restricted expression pattern has some potential advantages, such as the possibility of targeting intestinal stem cells without altering stem cell pools in other tissues. *OLFM4* was identified in cells of the myeloid lineage, and *OLFM4* RNA was observed in human colon, stomach, and bone marrow in addition to the small intestine (Zhang et al., 2002). No LacZ reporter gene expression was detected in these organs in our mouse model. The restricted expression pattern may reflect a more limited function of *Olfm4* in the mouse, raising the possibility that other Olfactomedin family members are coexpressed with *Lgr5* in other tissues.

In conclusion, the *Olfm4-IRES-eGFPCreERT2* allele described here provides a tool, separate from *Lgr5*, that can be used to further characterize intestinal stem cells.

EXPERIMENTAL PROCEDURES

Mice

Olfm4-IRES-eGFPCreERT2 KI mice were generated with the use of the KI construct as depicted in Figure 1C. The targeting construct (100 g) was linearized and transfected into male 129/Ola-derived IB10 ESCs by electroporation (800 V, 3 μF). Recombinant ESC clones expressing the neomycin gene were selected in medium supplemented with G418 (200 g/ml). Approximately 500 recombinant ESC clones were screened by Southern blotting. Positive clones were injected into C57BL/6 blastocysts with the use of standard procedures. The neomycin selection cassette was flanked by LoxP recombination sites and excised in vivo by crossing the mice with the PGK-Cre mouse stain (Lallemand et al., 1998). *Rosa26-LacZ* Cre reporter mice were obtained from The Jackson Laboratory. Eight-week-old mice were analyzed for eGFP signals or injected intraperitoneally with 200 μl tamoxifen in sunflower oil at 10 mg/ml. All procedures were performed in compliance with local animal welfare laws, guidelines, and policies.

Histology, In Situ Hybridization, Immunofluorescence Labeling, and Galactosidase Assay

For in situ hybridizations, tissues of mice were fixed in 4% paraformaldehyde (PFA), paraffin embedded, and sectioned at 3–6 mm. The generation of probes targeting *Lgr5* and *Olfm4* was previously described (Tian et al., 2011; van der Flier et al., 2009a). The protocols used for in vitro transcription and in situ hybridization were previously described (Gregorieff et al., 2005). Immunofluorescence sample preparation was performed according to Snippert et al. (2011). The eGFP signal was enhanced using an Alexa-488-coupled rabbit-anti-GFP antibody (Invitrogen; 1:1,000 1 hr at room temperature) diluted in PBS, 2% normal goat serum, and 0.1% Triton X-100. DNA was counterstained using ToPro-3 (1:1,000; Invitrogen) or 4′,6-DAPI.

For fluorescence imaging, cultures were fixed (2% PFA, overnight at 4°C), permeabilized (0.2% Triton X-100/PBS), blocked (2% goat serum/0.1% Tween 20/PBS), and incubated in Alexa-488-coupled rabbit-anti-GFP (Invitrogen; 1:1,000, 1 hr at room temperature). Fluorescence was detected with TOPRO-3 or DAPI counterstaining (Invitrogen). Images were captured using a SP5 confocal microscope (Leica Microsystems).

LacZ staining was performed as previously described (Barker et al., 2007). Five stretches of proximal intestine totaling >300 crypts were counted. In addition, the number of tracing events was counted at the most proximal part of intestines at different time points and normalized for the size of the area. At least two mice per time point were analyzed and the relative amount of tracings after 1 day was set at 100%.

Organoid Culture

Mouse organoids were established and maintained as described previously (Sato et al., 2009) from isolated crypts of the proximal small intestine (the first 4 cm).

SUPPLEMENTAL INFORMATION

Supplemental Information includes four figures and can be found with this article online at http://dx.doi.org/10.1016/j.stemcr.2014.05.018.

ACKNOWLEDGMENTS

We thank Hugo Snippert for analysis of the *Olfm4*^{null} animals and Valentina Sasselli for a critical reading of the manuscript. J.S. was supported by Cancer Genomics Centre II, Molecular Mechanisms.

Received: November 11, 2013
Revised: May 22, 2014
Accepted: May 23, 2014
Published: June 26, 2014

REFERENCES

Barker, N., van Es, J.H., Kuipers, J., Kujala, P., van den Born, M., Cozijnsen, M., Haegebarth, A., Korving, J., Begthel, H., Peters, P.J., and Clevers, H. (2007). Identification of stem cells in small intestine and colon by marker gene Lgr5. Nature *449*, 1003–1007.

Bjerknes, M., and Cheng, H. (2006). Intestinal epithelial stem cells and progenitors. Methods Enzymol. *419*, 337–383.

Buczacki, S.J., Zecchini, H.I., Nicholson, A.M., Russell, R., Vermeulen, L., Kemp, R., and Winton, D.J. (2013). Intestinal label-retaining cells are secretory precursors expressing Lgr5. Nature *495*, 65–69.

Chen, L., Li, H., Liu, W., Zhu, J., Zhao, X., Wright, E., Cao, L., Ding, I., and Rodgers, G.P. (2011). Olfactomedin 4 suppresses prostate cancer cell growth and metastasis via negative interaction with cathepsin D and SDF-1. Carcinogenesis *32*, 986–994.

Clevers, H. (2013). The intestinal crypt, a prototype stem cell compartment. Cell *154*, 274–284.

de Lau, W., Barker, N., Low, T.Y., Koo, B.K., Li, V.S., Teunissen, H., Kujala, P., Haegebarth, A., Peters, P.J., van de Wetering, M., et al. (2011). Lgr5 homologues associate with Wnt receptors and mediate R-spondin signalling. Nature *476*, 293–297.

Gregorieff, A., Pinto, D., Begthel, H., Destrée, O., Kielman, M., and Clevers, H. (2005). Expression pattern of Wnt signaling components in the adult intestine. Gastroenterology *129*, 626–638.

Inomata, H., Haraguchi, T., and Sasai, Y. (2008). Robust stability of the embryonic axial pattern requires a secreted scaffold for chordin degradation. Cell *134*, 854–865.

Itzkovitz, S., Lyubimova, A., Blat, I.C., Maynard, M., van Es, J., Lees, J., Jacks, T., Clevers, H., and van Oudenaarden, A. (2012). Single-molecule transcript counting of stem-cell markers in the mouse intestine. Nat. Cell Biol. *14*, 106–114.

Kemper, K., Prasetyanti, P.R., De Lau, W., Rodermond, H., Clevers, H., and Medema, J.P. (2012). Monoclonal antibodies against Lgr5 identify human colorectal cancer stem cells. Stem Cells *30*, 2378–2386.

Lallemand, Y., Luria, V., Haffner-Krausz, R., and Lonai, P. (1998). Maternally expressed PGK-Cre transgene as a tool for early and uniform activation of the Cre site-specific recombinase. Transgenic Res. *7*, 105–112.

Li, H., Rodriguez-Canales, J., Liu, W., Zhu, J., Hanson, J.C., Pack, S., Zhuang, Z., Emmert-Buck, M.R., and Rodgers, G.P. (2013). Deletion of the olfactomedin 4 gene is associated with progression of human prostate cancer. Am. J. Pathol. *183*, 1329–1338.

Liu, W., Yan, M., Liu, Y., Wang, R., Li, C., Deng, C., Singh, A., Coleman, W.G., Jr., and Rodgers, G.P. (2010). Olfactomedin 4 down-regulates innate immunity against Helicobacter pylori infection. Proc. Natl. Acad. Sci. USA *107*, 11056–11061.

Lopez-Garcia, C., Klein, A.M., Simons, B.D., and Winton, D.J. (2010). Intestinal stem cell replacement follows a pattern of neutral drift. Science *330*, 822–825.

Morita, H., Mazerbourg, S., Bouley, D.M., Luo, C.W., Kawamura, K., Kuwabara, Y., Baribault, H., Tian, H., and Hsueh, A.J. (2004). Neonatal lethality of LGR5 null mice is associated with ankyloglossia and gastrointestinal distension. Mol. Cell. Biol. *24*, 9736–9743.

Muñoz, J., Stange, D.E., Schepers, A.G., van de Wetering, M., Koo, B.K., Itzkovitz, S., Volckmann, R., Kung, K.S., Koster, J., Radulescu, S., et al. (2012). The Lgr5 intestinal stem cell signature: robust expression of proposed quiescent '+4' cell markers. EMBO J. *31*, 3079–3091.

Potten, C.S. (1977). Extreme sensitivity of some intestinal crypt cells to X and gamma irradiation. Nature *269*, 518–521.

Potten, C.S., Kovacs, L., and Hamilton, E. (1974). Continuous labelling studies on mouse skin and intestine. Cell Tissue Kinet. *7*, 271–283.

Powell, A.E., Wang, Y., Li, Y., Poulin, E.J., Means, A.L., Washington, M.K., Higginbotham, J.N., Juchheim, A., Prasad, N., Levy, S.E., et al. (2012). The pan-ErbB negative regulator Lrig1 is an intestinal stem cell marker that functions as a tumor suppressor. Cell *149*, 146–158.

Ritsma, L., Ellenbroek, S.I., Zomer, A., Snippert, H.J., de Sauvage, F.J., Simons, B.D., Clevers, H., and van Rheenen, J. (2014). Intestinal crypt homeostasis revealed at single-stem-cell level by in vivo live imaging. Nature *507*, 362–365.

Sato, T., Vries, R.G., Snippert, H.J., van de Wetering, M., Barker, N., Stange, D.E., van Es, J.H., Abo, A., Kujala, P., Peters, P.J., and Clevers, H. (2009). Single Lgr5 stem cells build crypt-villus structures in vitro without a mesenchymal niche. Nature *459*, 262–265.

Snippert, H.J., van der Flier, L.G., Sato, T., van Es, J.H., van den Born, M., Kroon-Veenboer, C., Barker, N., Klein, A.M., van Rheenen, J., Simons, B.D., and Clevers, H. (2010). Intestinal crypt homeostasis results from neutral competition between symmetrically dividing Lgr5 stem cells. Cell *143*, 134–144.

Snippert, H.J., Schepers, A.G., Delconte, G., Siersema, P.D., and Clevers, H. (2011). Slide preparation for single-cell-resolution imaging of fluorescent proteins in their three-dimensional near-native environment. Nat. Protoc. *6*, 1221–1228.

Soriano, P. (1999). Generalized lacZ expression with the ROSA26 Cre reporter strain. Nat. Genet. *21*, 70–71.

Takeda, N., Jain, R., LeBoeuf, M.R., Wang, Q., Lu, M.M., and Epstein, J.A. (2011). Interconversion between intestinal stem cell populations in distinct niches. Science *334*, 1420–1424.

Tian, H., Biehs, B., Warming, S., Leong, K.G., Rangell, L., Klein, O.D., and de Sauvage, F.J. (2011). A reserve stem cell population in small intestine renders Lgr5-positive cells dispensable. Nature *478*, 255–259.

van der Flier, L.G., and Clevers, H. (2009). Stem cells, self-renewal, and differentiation in the intestinal epithelium. Annu. Rev. Physiol. *71*, 241–260.

van der Flier, L.G., Haegebarth, A., Stange, D.E., van de Wetering, M., and Clevers, H. (2009a). OLFM4 is a robust marker for stem cells in human intestine and marks a subset of colorectal cancer cells. Gastroenterology *137*, 15–17.

van der Flier, L.G., van Gijn, M.E., Hatzis, P., Kujala, P., Haegebarth, A., Stange, D.E., Begthel, H., van den Born, M., Guryev, V., Oving, I., et al. (2009b). Transcription factor achaete scute-like 2 controls intestinal stem cell fate. Cell *136*, 903–912.

VanDussen, K.L., Carulli, A.J., Keeley, T.M., Patel, S.R., Puthoff, B.J., Magness, S.T., Tran, I.T., Maillard, I., Siebel, C., Kolterud, Å., et al. (2012). Notch signaling modulates proliferation and differentiation of intestinal crypt base columnar stem cells. Development *139*, 488–497.

Zhang, J., Liu, W.L., Tang, D.C., Chen, L., Wang, M., Pack, S.D., Zhuang, Z., and Rodgers, G.P. (2002). Identification and characterization of a novel member of olfactomedin-related protein family, hGC-1, expressed during myeloid lineage development. Gene *283*, 83–93.

Stem Cell Reports, Volume *3*

Supplemental Information

Robust Cre-Mediated Recombination in Small Intestinal Stem Cells Utilizing the *Olfm4* Locus

Jurian Schuijers, Laurens G. van der Flier, Johan van Es, and Hans Clevers

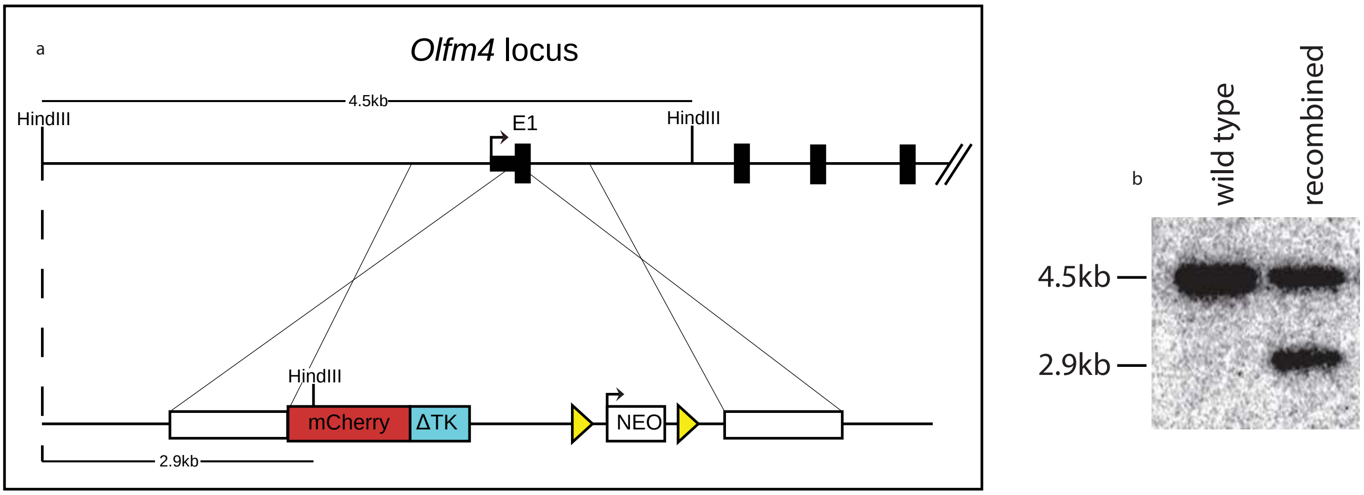
a
Olfm4 locus
4.5kb
HindIII
E1
HindIII
HindIII
mCherry
ΔTK
NEO
2.9kb
b
wild type
recombined
4.5kb
2.9kb

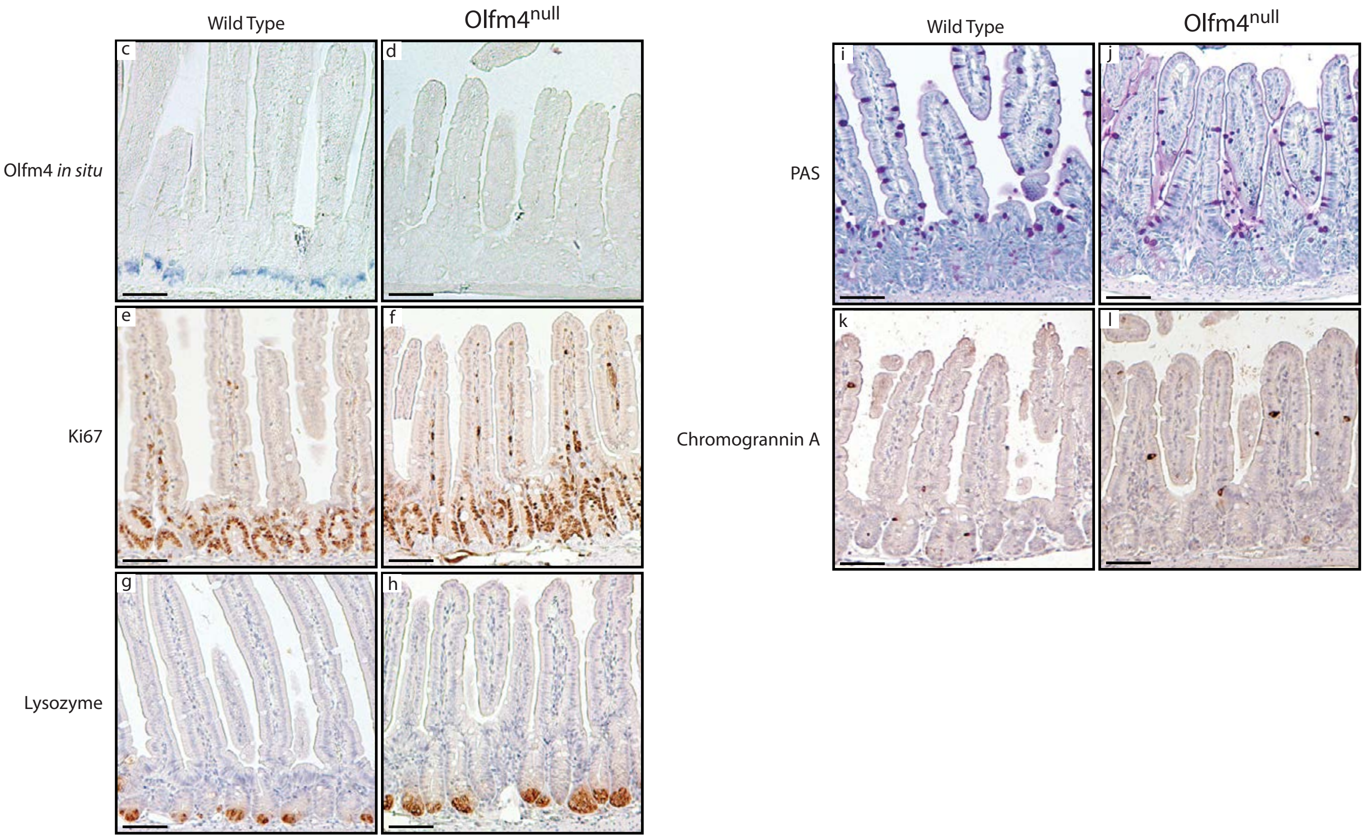
Wild Type
Olfm4null
Olfm4 *in situ*
Ki67
Lysozyme
c
d
e
f
g
h
Wild Type
Olfm4null
PAS
Chromogrannin A
i
j
k
l

Supplementary Figure 1. Olfm4^{null} animals show no intestinal phenotype. Supplementary to Figure 1.

A) Shows the targeting strategy for the generation of Olfm4-CherryΔtk knock-in allele. The CherryΔtk fusion is introduced in frame of the ATG in the first exon of the *Olfm4* locus. Neo, neomycin resistance cassette.

B) Southern blot of targeted mouse ES cells shows a homozygous wild-type allele in control lane 1 and a heterozygous-targeted allele in lane 2.

C,D) *In situ hybridization* probing for Olfm4 mRNA showing the complete loss of Olfm4 messenger in the Olfm4^{null} intestines.

E,F) Immunohistology staining for Ki67 normal proliferation in wild type and Olfm4^{null} intestines.

G,H) Immunohistology staining for lysozyme showing normal numbers and localization of Paneth cells in wild type and Olfm4^{null} intestines.

I,J) PAS staining showing normal numbers of goblet cells in wild type and Olfm4^{null} intestines.

K,L) Immunohistology staining for Chromogrannin A showing normal numbers of enteroendocrine cells in wild type and Olfm4^{null} intestines. Scale bars 100μM.

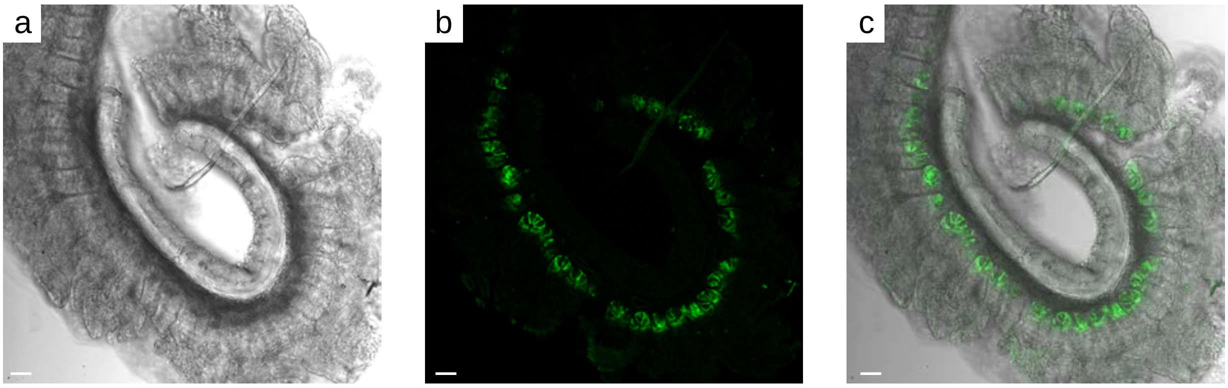
a
b
c

Supplementary Figure 2. *Olfm4-IRES-eGFPCreERT2* marks all the crypts of the small intestine. Supplementary to Figure 2.

A-C) Low magnification fluorescence microscopy showing complete penetrance of the *Olfm4-IRES-eGFPCreERT2* alleles in heterozygous animals. Scale bars 50µM.

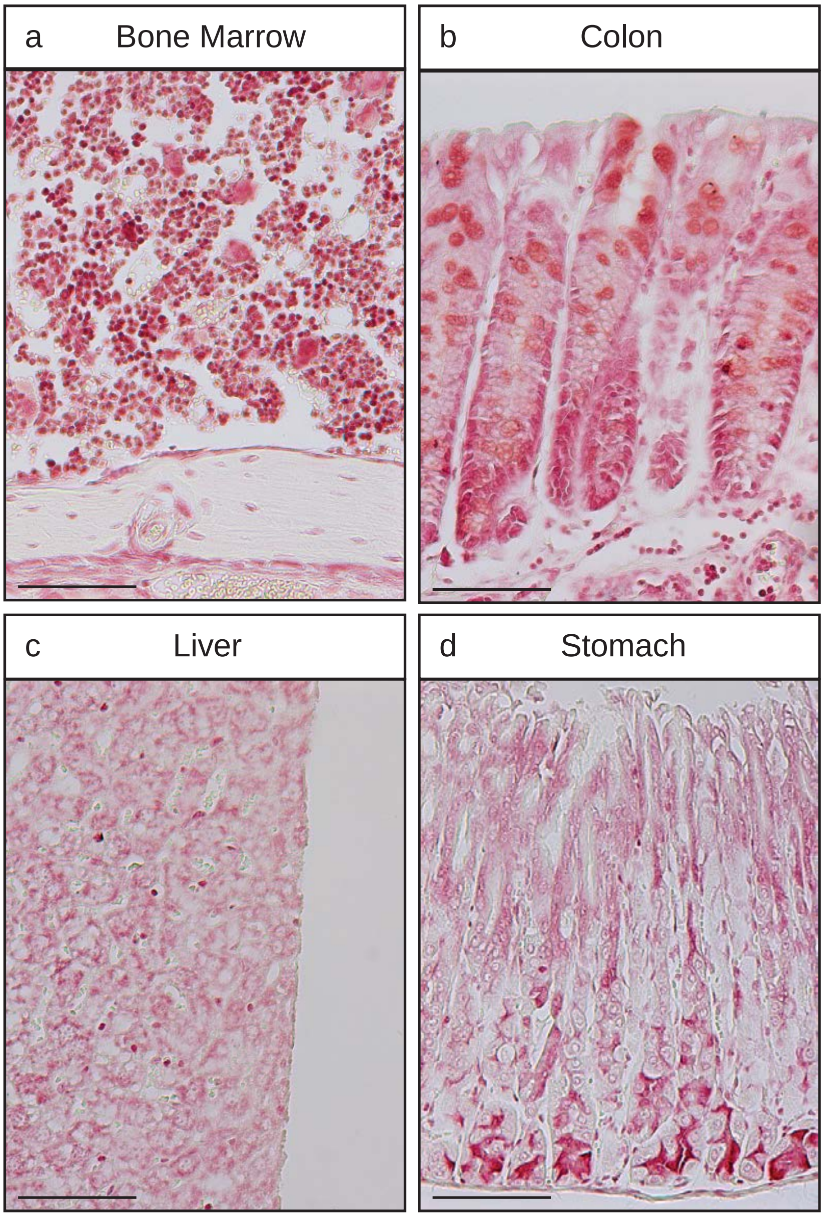
a
Bone Marrow
b
Colon
c
Liver
d
Stomach

Supplementary Figure 3. *Olfm4-IRES-eGFPCreERT2* is exclusively expressed in the small intestine. Supplementary to figure 3.

A-D) Immunohistochemistry showing representative pictures of the a) Bone Marrow, b) Colon, c) Liver and d) Stomach of tamoxifen induced heterozygous *Olfm4-IRES-eGFPCreERT2* 7 days after induction. Scale bars 50µm.

7days

+ tamoxifen | control

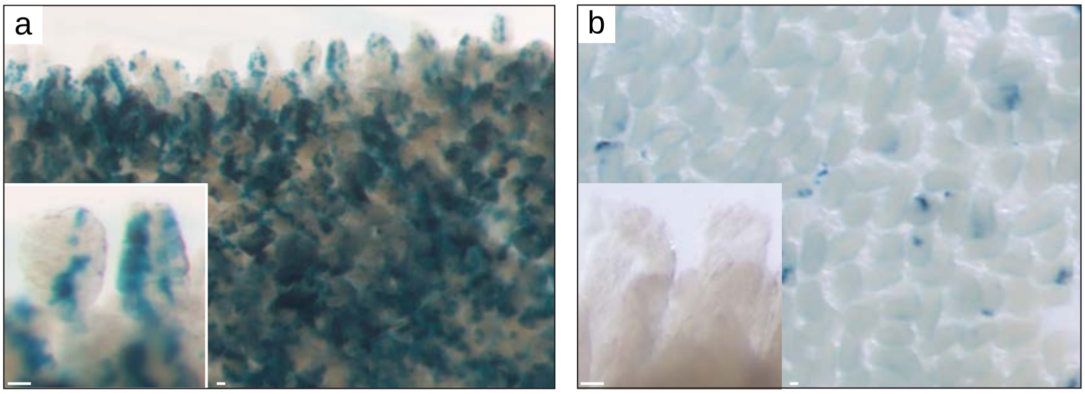

Supplementary Figure 4. Olfm4-IRES-eGFP-CreERT2 background induction. Supplemenary to figure 4.

A) Whole mount image showing LacZ staining of an *Olfm4-IRES-eGFPCreERT2* heterozygous animal 7 days after induction with tamoxifen.

B) Whole mount image showing LacZ staining of an *Olfm4-IRES-eGFPCreERT2* heterozygous animal without tamoxifen induction. Scale bars 50µM.
